# Functional Perspectives on Emotion, Behavior, and Cognition

**DOI:** 10.3390/bs3040536

**Published:** 2013-10-01

**Authors:** Heather C. Lench, Kathleen E. Darbor, Logan A. Berg

**Affiliations:** Department of Psychology, Texas A&M University, 4235 TAMU, College Station, TX 77843, USA; E-Mails: kedarbor@tamu.edu (K.D.); lberg86@gmail.com (L.B.)

**Keywords:** emotion, behavior, cognition, functional perspectives

## Abstract

This Editorial reviews the challenges and advantages posed by a functional perspective on the relationships among emotion, behavior, and cognition. We identify the core themes among the articles published as part of this Special Issue. The articles generally address two important questions: (1) are emotions functional and what is their impact on behavioral and cognitive processes, and (2) how do the interactions among emotion, cognition, and behavior play out in particular situations that present adaptive challenges? We also identify two core questions raised by the articles included in this Special Issue. Future research must address the extent to which emotions are best represented as discrete emotional constructs (e.g., anger, sadness, fear) *versus* emotions that vary along dimensions, such as valence and arousal. Functional perspectives would also be facilitated by identification of situations or environments that are likely to elicit particular emotions and reactions.

## 1. Introduction

The articles that compose this Special Issue tackle a critical and complex issue in modern psychology—the intersection of emotion with behavior and cognition. It has long been acknowledged that emotions, behavior and cognition represent core intrapsychic processes in humans. Indeed, these components are considered to be so fundamental that they are often referred to as the “ABCs of psychology”, for affect, behavior, and cognition [1]. Despite recognition that these components interact and exert powerful influences on one another, theories and evidence have traditionally been developed with a focus on only one component. While this focus has permitted greater understanding of emotion, behavior, and cognition within particular subareas of psychology, the proliferation of theories specific to one area has made it difficult to understand and predict reactions to events that occur across systems [2]. For example, the presence of a threat alters almost every aspect of experience and action, but most theoretical approaches only predict a subset of those reactions and it is often unclear how reactions might interact. The purpose of this Special Issue was to gather articles that utilize theoretical perspectives that transcend a focus on only one component. In this Editorial, we identify the main issues addressed in the individual articles and propose a set of additional questions for the field related to functional perspectives. Functional perspectives, which broadly propose that mental states and behaviors evolved to meet environmental demands, have the potential to unify our understanding of all three components as serving goals within particular environments. 

In a famous retrospective on autobiographical memory research, Allan Baddeley [2] prompted researchers to take a functional perspective. He suggested that, after an effect is demonstrated, researchers should ask themselves: “but what the hell is it for?” Asking this question about the function of processes prompts studies to identify how those processes impact an organism and allows prediction of how effects are likely to play out in different environments and situations. Such a perspective also prompts researchers to consider how processes influence the daily lives of people [3]. Effectively, the focus of inquiry changes from whether outcomes and actions are “correct” in a rational objective sense, to how the processes meet the challenges currently faced by the person. Even actions or biases that seem irrational or non-optimal at first glance might serve some broader need for adaptation. Optimism, for example, can reflect unrealistically positive illusions about the self, but such irrational cognitions can help motivate people to attain goals [4]. Although Baddeley’s comments were specific to research on memory, the functional perspective he endorses can allow for theory development with implications across areas and better understanding of the human mind.

The current Special Issue was intended to highlight theories and studies that apply a functional perspective to understand emotional, behavioral, and cognitive processes. The articles generally address two important questions: (1) are emotions functional and what is their impact on behavioral and cognitive processes, and (2) how do the interactions among emotion, cognition, and behavior play out in particular situations that present adaptive challenges?

## 2. Are Emotions Functional?

Weisfeld and Goetz [5] explore how a functional perspective could enhance empirical investigations of emotion. They use this perspective to integrate research on emotions across species and on the development of emotions in humans. They also present evidence that emotions correlate with behavior and physiology. Such a correlation would be expected if emotions arise as part of a coordinated response to environmental challenges, and would allow for fast and effective reactions to address those challenges. Their article delineates the contribution that functional perspectives can provide to the study of specific components, such as emotion, and opens new lines of inquiry for future research. 

Trimmer, Paul, Mendl, McNamara, and Houston [6] present a model to understand the contribution that moods, rather than emotions, make to adaptive decision making. They argue that moods can be used as information about the relative costs and benefits of preparing to take action. Taking an evolutionary perspective, they argue that moods allow people to quickly determine the behavior that would be appropriate—to approach desirable stimuli such as prey or to avoid undesirable stimuli such as a predator. In this model, then, emotional processes provide information that is critical to the survival and flourishing of humans. 

Bench and Lench [7] provide a functional perspective on a particular emotion – boredom. They propose that boredom is a functional discrete emotion with important consequences for goal pursuit, namely in the form of prompting pursuit of alternative goals and experiences with greater reward and risk. They explore potential distinct effects of boredom on behavior, cognition, experience, and physiology, making predictions about how boredom will impact cognition and behavior that can serve as the basis for future empirical work. This proposal serves as a potential framework for the development of functional accounts of other emotions that have been under researched.

Together, these papers present functional approaches to the study of emotion as an adaptation to environmental challenges, either as information to other systems or as factors that broadly influence cognitive and behavioral responses. They also identify important questions for future empirical study. Perhaps the most important among these is whether the traditional distinction held between emotion and cognition is worthwhile. On one hand, all of the papers present evidence of the far-reaching consequences of emotional experiences on cognition and behavior. On the other hand, the fact that emotion and cognition are so strongly integrated might indicate that they cannot be easily parsed. Taking a functional perspective might offer one avenue through which emotional and cognitive theories and predictions could be either combined or, when different predictions are made, compared.

## 3. What is the Impact of the Interdependence of Systems in Particular Situations?

One benefit of a functional perspective is that it encourages researchers to consider how processes play out in daily life [2]. Given that emotions, behavior, and cognition are interdependent, how does their interaction impact particular situations that pose adaptational problems? Two manuscripts addressed this question by examining how the interaction among components impacts situations common to daily experience. Sass, Fetz, Oetken, Habel, and Heim [8] focused on how emotions relate to executive functioning, which broadly reflects the ability to control and regulate cognition and behavior. They present evidence that emotional processes likely play a role in tasks that measure executive functioning. Keren, Boyer, Mort and Eilam [9] also focused on a commonly encountered adaptational challenge in the need to identify and address threats. They present evidence that idiosyncratic behaviors function to reduce anxiety in threatening environments. Using a functional perspective, they predicted and found that people react to threat by exploring multiple potential actions and taking more time to consider their responses. Although outside the scope of their investigation, it is likely that such an approach to threatening situations would improve the chances of surviving a threat. 

Two articles examined the impact of interactions among systems when there is less than optimal functioning in one of the components. The studies reported in these articles reveal that when there is an issue in emotional or cognitive functioning, it has far-reaching implications for cognition, and potentially behavior. John and DiLalla [10] examined the influence of emotional biases on reports of child peer victimization from children and their parents. They found that parents and children who had difficulty recognizing emotional expressions were more likely to disagree in their reports of victimization. This finding has implications for understanding the consequences of emotional processes in threatening situations, as well as how emotions impact perceptions of the self and others. Radaelli, Benedetti, Cavallaro, Colombo, and Smeraldi’s [11] findings suggest that responses to emotional material are related to cognitive delusions. Schizophrenic patients with psychotic symptoms demonstrated biases in processing self-generated material and had difficulty evaluating their mental experiences. 

Together, these articles provide evidence that the interdependence among emotion, cognition, and behavior that is predicted by functional accounts have implications for daily experience, both normal and abnormal. The findings reported in these studies suggest that a functional approach might offer unique insight into traditional treatments and therapies that attempt to alter emotion, cognition, or behavior to improve quality of life, as well as suggest new avenues for intervention. 

## 4. Conclusions

The articles in this Special Issue provide perspectives on how functional approaches can be applied to the study of the interactions among emotion, cognition, and behavior. The articles also highlight areas for future investigation. One question raised by the articles as a whole is whether emotions represent discrete organized reactions to situational circumstances, such as anger in response to obstacles to goal attainment, *versus* reactions that vary along some dimension, such as valence and arousal. Additional research is needed to identify whether discrete emotions, such as anger, sadness, and fear, represent discrete emotional states with a unique evolutionary history. Alternatively, negative emotions might be separable from positive emotions, with discrete emotions varying only in terms of how they are culturally represented. 

Another core question remains the extent to which observed changes in emotion, cognition, and behavior, and the correlations among them, are related to specific environmental conditions and produce positive outcomes in those conditions. It is remarkably easy to start with an observed effect and weave a narrative about how the effect might have developed in early evolutionary history. Those narratives often risk simply reflecting our current cultural stereotypes and understanding and are notoriously difficult to test empirically. For functional accounts to be compelling, it is critical that predictions derived from those accounts be ultimately tested [12]. To do so, researchers must identify the environmental challenges faced by early humans and use those environmental constraints to make predictions about the emotional, cognitive, and behavioral reactions that would allow adaptation to the challenges. 

Each article in this Special Issue presents a facet of how a functional approach can integrate disparate theories and empirical findings and offers the potential to transcend typical disciplinary boundaries to predict and understand human reactions. 
